# Editorial: Quantitative imaging methods and analysis in Alzheimer's disease assessment

**DOI:** 10.3389/fnagi.2023.1340877

**Published:** 2024-01-09

**Authors:** Stephanos Leandrou, Deqiang Qiu, Constantino Carlos Reyes-Aldasoro

**Affiliations:** ^1^European University Cyprus, Engomi, Cyprus; ^2^Department of Radiology and Imaging Sciences, School of Medicine, Emory University, Atlanta, GA, United States; ^3^Senior Lecturer in Biomedical Image Analysis giCentre, Department of Computer Science, School of Science and Technology City, University of London, London, United Kingdom

**Keywords:** Alzheimer's disease, quantitative imaging, machine learning, PET, CSF, gene

Alzheimer's disease (AD) is a complex condition gradually eroding memory and cognitive abilities. While cognitive tests aid diagnosis, their limitations include an inability to detect subtle early-stage changes, subjective interpretation influenced by various factors, and inconsistent predictive accuracy. Imaging techniques play a pivotal role in AD detection by revealing structural and functional brain changes associated with the disease. Quantitative MRI highlights brain atrophy, ruling out other causes, while PET scans visualize hallmark proteins like beta-amyloid and tau. Cerebrospinal fluid (CSF) analysis assesses biomarkers linked to AD, notably Aβ42 and tau proteins. The APOE gene variants, specifically ε4, influence AD susceptibility. EEG, measuring brain electrical activity, offers supplementary information but isn't a primary diagnostic tool.

The goal of this Research Topic was to investigate the impact of quantitative imaging in the assessment of AD by using structural MRI or molecular neuroimaging such as PET as results from cognitive tests are not sufficient to accurately and reliably make the diagnosis of AD. Also, it evaluated the performance of deep learning methods in the classification and prediction of AD. Early detection is crucial for effective intervention and treatment.

- The study “*Piecing it together: atrophy profiles of hippocampal subfields relate to cognitive impairment along the Alzheimer's disease spectrum*,” investigated the relationship between AD and hippocampal subfield-specific neurodegeneration, aiming to enhance prognostic procedures. The findings suggest that considering subfield-specific hippocampal degeneration, along with cognitive assessments, could offer a sensitive prognostic approach for tracking disease trajectories in individuals on the AD spectrum (Christopher-Hayes et al.).
- The study “*A cross-sectional study of explainable machine learning in Alzheimer's disease: diagnostic classification using MR radiomic features”* focused on improving the assessment of AD by addressing the limitations of cognitive tests and late-stage brain atrophy detection through qualitative imaging. The researchers used Machine Learning (ML) methods, particularly XGBoost, to analyze radiomic features from the entorhinal cortex and hippocampus. The use of explainable ML approaches, such as SHAP (SHapley Additive exPlanations), demonstrated the potential for earlier AD diagnosis, better disease management, and the development of novel treatment strategies (Leandrou et al.).
- The study “Early-stage mapping of macromolecular content in APP NL-F mouse model of Alzheimer's disease using nuclear overhauser effect (NOE) MRI” explored the potential of Nuclear Overhauser enhancement (NOE) MRI as a non-invasive method for early detection of AD. NOE MRI provides contrast sensitive to lipid and protein content in the brain, which are known to be altered in AD pathology. Using template-based analyses and optimizing parameters for NOE specificity, the researchers detected changes in lipids and proteins in an AD mouse model in the hippocampus, hypothalamus, entorhinal cortex, and fimbria, indicating disruptions in the phospholipid bilayer of cell membranes in both gray and white matter regions. The findings suggest that NOE MRI could be a valuable tool for monitoring early-stage changes in lipid-mediated metabolism in AD and potentially other disorders, offering high spatial resolution (Swain et al.).
- The study “*Bayesian workflow for the investigation of hierarchical classification models from Tau-PET and structural MRI Data across the Alzheimer's disease spectrum”* addressed the challenges of early-stage AD diagnosis and explores the role of tau neurofibrillary tangles in brain networks. Using a Bayesian workflow and data from a longitudinal cohort, the researchers developed hierarchical multinomial logistic regression models based on tau-PET and structural MRI data. The findings suggest that considering hierarchical models encompassing ROI and network-level heterogeneity improves predictive accuracy in assessing AD using tau-PET and structural MRI data (Belasso et al.).
- The study “*A holo-spectral EEG analysis provides early detection of cognitive decline and predicts progression to Alzheimer's disease,”* aimed to differentiate individuals with MCI and AD from cognitively normal (CN) individuals, as well as predict the progression from MCI to AD using a newly developed technique called Holo-Hilbert Spectral Analysis (HHSA) applied to resting state EEG (rsEEG). The HHSA analysis revealed distinctive patterns in amplitude modulation (AM) power across frequency bands in MCI and various AD stages compared to CN controls. ML algorithms successfully discriminated between groups with good sensitivity and specificity in cross-sectional analysis. In a longitudinal follow-up of MCI patients, HHSA identified significant differences in AM power between stable and converted MCI groups. The study suggests that integrating HHSA into EEG signals, combined with ML, holds promise for differentiating cognitive states and predicting progression in individuals with MCI (Chu et al.).
- The study “*APOE* ε*4 accelerates the longitudinal cerebral atrophy in OASIS-3 elders without dementia at enrolment”* investigated the modulation of the APOE gene on the trajectory of cerebral atrophy during the conversion from CN to dementia. Using a voxel-wise whole-brain perspective and data from the OASIS-3 neuroimaging cohort, the researchers applied a linear mixed-effects model to detect cerebrum regions with non-linear atrophic trajectories influenced by AD conversion and to examine the impact of APOE variants on these trajectories. Results showed that participants transitioning from CN to dementia exhibited faster quadratically accelerated atrophy in bilateral hippocampi compared to persistent CN individuals. The study contributes to understanding how APOE ε4 influences hippocampal atrophy acceleration and the progression from normal cognition to dementia (Huang et al.).
- The study “*Brain atrophy in non-demented individuals in a long term longitudinal study from two independent cohorts*” investigated the longitudinal atrophy rates in individuals with AD, those with increased phosphorylated Tau at Thr181 (pTau181) without amyloidopathy (A–T+N±), and biomarker-negative non-demented subjects. Using data from two independent cohorts, the results showed that individuals with increased pTau181 levels without amyloidopathy (A–T + N±) did not exhibit a unique longitudinal atrophy pattern compared to AD or biomarker-negative subjects. In contrast, those with both increased pTau181 and amyloidopathy (A+T + N±) showed marked longitudinal atrophy in the temporal lobe. The study suggests that increased pTau181 levels without concomitant amyloidopathy may not indicate a neurodegenerative disorder, as there was no significant atrophy rate compared to controls in these individuals (Haas et al.).

Future work entails addressing resolution limitations in imaging, enhancing quantitative assessment for precise analysis, understanding the disease's heterogeneity, and integrating diverse data sources. Identifying reliable imaging biomarkers specific to AD is crucial, requiring standardization in protocols and analysis methods across studies and clinical settings. Additionally, interpreting imaging findings accurately amid various health conditions and age-related changes is pivotal. Overcoming these challenges demands collaborative efforts, technological advancements, standardized protocols, and a deeper understanding of AD's intricate mechanisms.

## Author contributions

SL: Writing – original draft. CR-A: Contributed to manuscript preparation and revision. DQ: Read and approved the final submitted version. All authors discussed the results and commented on the manuscript.

